# Comment on “Prevalence and Risk Factors for Diabetic Lower Limb Amputation: A Clinic-Based Case Control Study”

**DOI:** 10.1155/2017/6015326

**Published:** 2017-10-17

**Authors:** M. Bakhtiyari, M. A. Mansournia

**Affiliations:** ^1^Prevention of Metabolic Disorders Research Center, Research Institute for Endocrine Sciences, Shahid Beheshti University of Medical Sciences, Tehran, Iran; ^2^Department of Epidemiology and Biostatistics, School of Public Health, Tehran University of Medical Sciences, Tehran, Iran

We read with interest the study conducted by Rodrigues et al. [1] published in June 2016.

In this study, the studied sample has been selected from the target population (at Northeastern Australia's Townsville Hospital High Risk Foot Clinic) without considering the exposure (risk factors of limb amputations amongst diabetic patients) and the outcome (amputation). This type of study design is compatible with the cross-sectional study design, and the number of participants with and without the outcome is not prespecified in the design of this kind of study [2].

Moreover, in the current study, the samples are not randomly selected, so they are not good representatives of the target population of the study and the estimated prevalence can be generalized only for the patients referred to the mentioned clinic. The authors of the current study have considered the study as a case-control one both in the title and in the context, but sampling in a case-control study is based on the outcome status and the number of the participants with and without the outcome (amputation) is fixed and known in advance in the design of the study. Moreover, it is worth mentioning that estimation of the prevalence is not possible in case-control studies. The title of the study may be confusing since the study is not a true case-control study; hence, there is a need to amend this case [3].

Another point is that in the discussion some comparisons were made between the estimation of prevalence in the present study and those of other studies. However, it is usually not recommended to generalize the results of studies to populations that are different from those in which the study was performed, as the outcomes may be very different in different populations. The populations should be matched (i.e., propensity matched) or somehow a standardization or reweighting should occur to manage the issues of nonblinded nonrandomized data [3, 4]. The proper labeling of the design of the study can contribute to better understanding of it.
